# Why are disorders of gut–brain interaction (DGBI) often food-related? Duodenal eosinophils and mast cells, small intestinal bacteria, food allergy and altered food intake in functional dyspepsia and the irritable bowel syndrome: a new paradigm

**DOI:** 10.1007/s00535-025-02268-2

**Published:** 2025-06-30

**Authors:** Nicholas J. Talley, Kerith Duncanson, Georgina M. Williams

**Affiliations:** School of Medicine and Public Health, College of Health, Medicine and Wellbeing, University of Newcastle, Newcastle, Australia

**Keywords:** DGBI, IBS, FD, Diet, Microbiome, Immunology

## Abstract

The underlying causes of irritable bowel syndrome (IBS) and functional dyspepsia (FD) have remained largely elusive, but emerging data suggest immune activation and loss of small intestinal homeostasis may explain a major subgroup. FD and IBS symptoms often overlap and may occur early in the post-prandial period, suggesting the origin of symptoms may be much higher in gastrointestinal tract than colon. There is strong evidence low-grade duodenal inflammation, comprising eosinophils and/or mast cells associated with increased permeability, is present at least in a major subset with FD and IBS. This hypothesis is further supported by evidence of circulating increased small intestinal homing T cells and altered duodenal microbiota. We hypothesize a major etiologic pathway whereby interaction of food with intestinal bacteria switches on small intestinal immune activation in FD and IBS leading to presentation of antigens to the mucosa. While the low FODMAP diet provides symptom relief in both IBS and FD, this diet notably also reduces common food protein antigens (e.g., wheat, milk, soy) and urinary histamine levels. The obvious but often overlooked fact that food ingestion usually requires the act of eating adds nuance to determining whether food components or eating itself induces symptoms and that both need to be considered in DGBI in clinical practice. The exciting observations about subtle inflammation in DGBIs offer hope for new diagnostic biomarkers, and if considered in the context of altered dietary patterns and validated against symptom responses, will pave the way for novel DGBI treatment options.

## Introduction

Disorders of gut–brain interactions (DGBIs) including irritable bowel syndrome (IBS) and functional dyspepsia (FD) are highly prevalent, unexplained conditions that substantially impact the quality of life of over one-third of the community from children to adults [1–3]. IBS is characterized by chronic abdominal pain and disturbed defecation, while FD is characterized by chronic early satiety, post-prandial fullness, and epigastric pain [1–3]. DGBIs are the most common disorders diagnosed by gastroenterologists, impair quality of life (e.g., affecting sleep, personal relationships and sexual functioning), often result in unnecessary surgeries, are an important cause of mental ill health and permanent disability, and incur enormous direct and indirect costs [4]. IBS and FD may develop after infectious gastroenteritis, antibiotic use or pet exposure, implicating the microbiota in the etiopathogenesis [5, 6]. Anxiety, depression and sleep disturbances often develop after DGBI symptom onset, implicating gut–brain pathways [7–9].

It is well-established that FD and IBS overlap more than expected by chance, suggesting they share a common underlying pathogenesis, at least in a subset [10]. The symptoms of FD are considered to arise from gastroduodenal sensory and motor disturbances, whereas in IBS, the focus has traditionally been on sensory–motor disturbances in the colon [1–3]. While the underlying causes of these disturbances has remained largely mysterious, emerging data suggest immune activation and loss of small intestinal homeostasis driven by diet and the microbiota may explain a major subgroup with FD or IBS as discussed here.

## Food-induced symptoms often occur soon after eating in FD and IBS

The majority of patients with FD have meal-induced symptoms, which define the post-prandial distress syndrome (post-prandial fullness and/or inability to finish a normal sized meal) [2, 3]. When symptoms are prospectively measured after meal ingestion, the peak symptom response occurs 30 min later, implicating stomach or small intestine in the origin of symptoms. What is much less appreciated is that in IBS, symptoms often also occur early in the post-prandial period [11–14]. Using food and symptom diaries, pain in IBS has been more closely linked to eating, not defecation [14]. In a New Zealand study, IBS subjects often had meal-induced symptoms and were more likely to experience this pattern than those with other functional bowel disorders by Rome criteria [12]. Further, in food and symptom diaries of 9710 individuals (~ 70% with IBS), respondents associated certain foods with symptom induction including bloating, flatulence, pain and diarrhea within one to two hours post ingestion [15]. Foods associated with symptoms varied in composition and included bloating after seaweed (allergen and salicylates), chips and meats (dietary fats), onions and garlics (fructans), condiments (salicylates, amines, and fructans), and tomato (salicylates) intake, suggesting that diverse mechanism of action could be implicated. Flatulence was commonly reported after eating onion and garlic (fructans), pain after foods containing corn syrup (fructose) and diarrhea from coffee or alcohol (intestinal irritants). These observations are important as they suggest food-induced symptoms in IBS, like in FD, cannot arise from colonic disease in all cases because there is insufficient time for chyme to reach the colon based on the timing of symptom induction (except in rare circumstances of exceptionally rapid small intestinal transit).

Separate to, but resulting from these early symptom responses to food, the psychological impact of symptoms in response to food and eating is gaining attention. Altered dietary patterns necessitated by exclusion diets that are trialed to mitigate GI symptoms can result in symptom relief but can also perpetuate long-term restrictive eating practices [16]. There is emerging research and considerable debate about differentiating food restriction and avoidance for the purpose of managing DGBI symptoms and overt eating disorders [17]. While fear of aversive consequences is a strong driver of altered food intake in DGBI, this is potentially attributable to managing the GI condition, rather than a maladaptive dietary behavior. Differentiating DGBI-initiated dietary alterations from maladaptive eating behaviors that develop into a mental health condition or co-exist with a DGBI is an important facet of DGBI medical and dietetic management that requires new diagnostic tools and clinical management guidelines.

## Low-grade inflammation and immune activation in the duodenum in FD and IBS

The hypothesis that interactions between food and small intestine are relevant in pathogenesis of DGBI is supported by several lines of evidence that implicate small intestinal pathology in both FD and IBS. Population-based studies have shown FD and IBS are both significantly associated with atopic eosinophilic diseases, such as asthma, allergic rhinitis and eczema [18–20]. Talley et al. discovered in 2007 that there was increased duodenal eosinophilia in FD diagnosed in a randomly selected community sample compared with healthy controls from the same population, with evidence of eosinophil activation as shown by increased eosinophil degranulation [21]. Subsequent work from Australia [22] and internationally has confirmed these observations [23, 24]. There is also evidence that symptom severity correlates with increased duodenal eosinophils in FD, and if eosinophils are reduced, this correlates with symptom improvement [24]. This increase in eosinophils has been associated with altered metabolite profiles, with patients with FD and eosinophilia found to have altered lipid metabolism and increased markers of oxidative stress compared to individuals with FD but without eosinophilia [25]. Further, increased duodenal permeability has been observed in FD [22, 24]. Mast cells are also increased in the duodenum in IBS, indicating immune involvement, but in the fasting state, eosinophils are not usually increased in IBS unless there is IBS and FD overlap [26, 27].

Conversely, the elevated duodenal eosinophil counts that underpin the ‘inflammatory’ model of DGBI are far from universal. A recent systematic review highlights that controls have highly variable eosinophil counts that frequently overlap with those observed in patient cohorts [23]. A 2024 publication has reported a standardized protocol for assessing duodenal histology, and it is recommended that future research utilizes these standards to ensure consistency and comparability [28]. It is also notable that sampling for eosinophil counts in all these studies was done while the patient is fasting, yet symptoms in FD and IBS usually occur after meals, so duodenal low-grade inflammation may have been missed if food plays a key role in the pathogenesis.

In addition to low-grade small intestinal inflammation in at least a subset with FD and IBS, it has been observed that there are increased small intestinal homing T cells in serum in FD, objectively confirming the presence of small intestinal inflammation [29, 30].

## What could explain meal-related symptoms and duodenal inflammation in FD and IBS?

The etiology of symptoms and low-grade duodenal inflammation with increased eosinophils and mast cells in FD and IBS is likely to be multifactorial. It is well-established that acute gastrointestinal infections can lead to post-infectious IBS or FD (or both) at least in approximately 10% of DGBI cases (which may be an underestimate as identifying infection relies often on self-report) [31–33]. A recent publication reported that individuals with post-infectious IBS were found to have increased numbers of neutrophils and T helper 17 cells, higher interleukin 7 levels, and widened epithelial cell gaps compared to individuals with non-post-infectious IBS [34], suggesting gastrointestinal infection may lead to lasting changes in the immune and structural profile of the gut, with subsequent symptoms.

These findings are consistent with a 2021 study that reported IgE immune reactions to 'superantigens' could be responsible for persistence of food intolerance after a gastrointestinal infection had cleared [35]. Visceral hypersensitivity was proposed as the mechanism by which IBS type symptoms subsequently reoccurred when the dietary antigen was consumed again, suggesting the immune reaction was localized to the gastrointestinal tract [35].

Increased intestinal permeability from drugs or acute stress could also induce an intestinal inflammatory cascade [22, 36]. Smoking exposes the intestinal tract to multiple toxins and is a risk factor for both FD and IBS with diarrhea [37]. In this review, we will focus on two possible etiologic factors that may switch on small intestinal immune activation and lead to loss of homeostasis, namely food antigens and the interaction of food with intestinal bacteria.

## Evidence for atypical food allergy in IBS and FD

Classic IgE-mediated food allergy in IBS (e.g., to peanut, shellfish) is considered rare, and testing for food allergy in patients with IBS unless there is associated urticaria is not routine. Until recently, there has been little interest in the idea of eliminating antigenic foods in treating IBS and some disbelief atypical food allergy might play a role.

Major interest in the role of food elimination diets in IBS has followed the landmark randomized controlled trial diet studies showing a low fermentable carbohydrate (low FODMAP) diet reduced symptoms in 50–70% of patients with IBS versus a control diet [38]. A low FODMAP diet has been postulated to be efficacious primarily because it reduces colonic gas production from bacterial fermentation as well as reducing intestinal fluid secretion, thereby reducing signaling from visceral hypersensitivity [39]. However, this hypothesis poorly explains why a low FODMAP diet also improves extra-intestinal symptoms like fatigue in IBS or fibromyalgia, suggesting other mechanisms such as reducing immune activation may be impacted by the diet change [40]. Furthermore, a low FODMAP diet does not treat IBS, instead it is a diagnostic tool to identify food ‘triggers’ in sensitive individuals which can be used to determine long-term dietary management plans for symptom relief [41]. Colonic bacterial fermentation also doesn’t adequately explain why IBS symptoms often occur early after eating, or why a low FODMAP diet is also efficacious in FD, which has been shown in recent studies [42, 43].

Further, the exact role of FODMAPs remains to be clarified. Clevers et al. recently observed no association between FODMAP intake and self-reported GI symptoms in a cohort of 9710 participants. However, individuals reported symptoms of bloating and gas within 1–2 h of onion and garlic intake [15]. This finding is unexpected as garlic and onion are high in the FODMAP fructan, an oligosaccharide not absorbed in the small intestine and therefore suggested to only induce symptoms in the large intestine. This supports work by Masuy et al. who found administration of fructans intragastrically resulted in changes to gastric motility, gastrointestinal and extra-intestinal symptom onset within 3 h of onset in individuals with IBS [44].

Food antigens prevalent in IgE food allergy are reduced on the low FODMAP diet. For example, a low FODMAP diet reduces foods high in dietary fructans and galacto-oligosaccharides including wheat, soy, and nuts (containing food antigens gluten, gliadin, soy protein and tree nut) and limits foods high in lactose (milk products), thus reducing milk protein (casein and whey) intake. If these food antigens are responsible for gastrointestinal symptoms in at least in a subset of individuals with DGBI, symptom improvement may be due to reduced antigen intake and an immune threshold rather than complete exclusion as in typical food allergy. Mechanistic studies to determine whether non-FODMAP components in these foods may be implicated in symptom response on a low FODMAP diet are needed.

An example of a common food antigen in IgE food allergy is milk, which was recently implicated in symptom response in FD. A cohort study found that restricting milk for four weeks improved symptom scores in patients with FD compared to patients who received standard medical therapy for FD [45]. It should be noted that dietary intake beyond the inclusion or exclusion of milk was not assessed in both groups limiting adoptability of the findings [45].

This evidence that milk exclusion may provide symptom relief in FD is of interest as milk contains both lactose (FODMAP) and milk protein (casein or whey), both dietary components associated with gastrointestinal symptoms. In eosinophilic esophagitis, exclusion of milk for 6 weeks is associated with improved eosinophilia and symptoms [46]. Another study reported that a 6-week amino acid-based elemental diet in histologically active eosinophilic gastritis or duodenitis induced complete histologic remission in all subjects (*n* = 15) in stomach and duodenum, and improved symptomatic, quality-of-life, and molecular parameters [47]. These findings and disease recurrence with food trigger reintroduction support a dominant role for food allergens in disease pathogenesis [47]. While these eosinophilic conditions do not necessarily translate directly to the DGBI context, they do emphasize the need for carefully designed dietary intervention studies that explore both symptomatic and immunopathologic responses to food antigen exclusion in DGBI to better understand mechanisms of food-induced symptoms.

Other evidence implicating food antigens inducing an immune response that is not IgE mediated is emerging. A randomized trial providing an elimination diet based on foods with raised serum IgG antibodies versus a sham diet resulted in a modest symptom score benefit in IBS of 10% [48]. More impressive results were obtained in a recent randomized trial in which individuals with IBS followed an elimination diet based on individual IgG assay results. A significantly higher proportion of participants (60%) following the IgG-guided diet experienced meaningful symptom improvement (> 30% reduction in symptoms) compared to controls (42%). Notably, the most commonly excluded foods were wheat, egg, and cows milk [49].

Natural food chemicals including salicylates, amines, and glutamate are hypothesized to induce gastrointestinal symptoms via neuro-excitability and inflammatory pathways. However, mechanistic data are lacking. Observational data from Australia found that IBS participants were more likely to report extra-intestinal symptoms than healthy controls, and that ~ 30% of participants with IBS associated these symptoms, as well as gastrointestinal symptoms with food chemical intake [50]. A systematic review by the same authors concluded that evidence for the role of food chemicals in gastrointestinal symptoms is lacking. However, in other immune-mediated conditions including urticaria and aspirin exacerbated respiratory disease, a diet low in histamine or salicylate, respectively, may be useful [51].

## Direct evidence for acute food-induced duodenal immune activation in IBS and FD

Direct evidence for food antigen-induced symptoms in IBS is emerging. Elegant studies have applied confocal laser endomicroscopy in real-time changes in the duodenum after the instillation of food challenges on the duodenal mucosa [52, 53]. In IBS, four duodenal challenges to five common food antigen (wheat, soy, egg, milk, and yeast) were performed in one study, and it was observed over 50% had almost immediate disruption of the intestinal barrier with eosinophil degranulation as confirmed by increased eosinophil cationic protein in duodenal fluid [52]. In FD, very similar preliminary results have been presented from the Leuven group in terms of a 6-food elimination diet-reducing symptoms [54].

Wheat was the most common food implicated in IBS on confocal studies [52, 53]. Consistent with this, novel work from Australia has observed lamina propria mononuclear cells in response to gliadin but not gluten, provoking a duodenal effector Th2-like response in FD, implicating this food antigen in driving immune activation in a subset of patients with FD [55]. In 77 patients with FD and self-reported symptom improvement on a gluten-free diet, 6% had symptom reinduction with blinded gluten challenge [56]. Notably in this cohort, extra-intestinal symptoms were significantly improved in patients with FD on a gluten-free diet [56]. Non-celiac wheat sensitivity is a topical area of research in DGBI with clinical trial results marred by high nocebo responses [57] often due to a reliance on self-reported symptom response as a primary outcome. The overlap between fructan (FODMAP) and gluten (protein) components of wheat has also complicated dietary research although one study found no effect of gluten ingestion on gastrointestinal symptoms or markers of colonic epithelial barrier in IBS patients on a low FODMAP diet [58]. There is a lack of well-designed clinical trials examining both symptom and the immune response to food components in both IBS and FD and thus it is imperative that dietary intervention studies, carefully designed to control for all food components suspected to trigger symptom responses in DGBI, are undertaken.

## Coming full circle: some with IBS may have IgE mediated disease after all

True IgE-related food allergy might also play a more important local role in IBS and FD than has been appreciated. In elegant work in mice, a colonic infection broke oral tolerance increasing colonic permeability [35]. This in turn led to an adaptive immune response to food antigen with activated mast cells (primed with allergen specific IgE) releasing histamine and stimulating colonic splanchnic nerves. Further, injection of food antigens (wheat, gluten, soy, milk) into the rectosigmoid of adults with IBS was shown to induce a local mucosal reaction with mast cell degranulation in all 12 IBS cases and 2 of 8 controls [35].

Another example of IBS-like symptoms being induced by an IgE food allergic response is the alpha-gal syndrome [59]. Here a tick bite exposes the host to a foreign carbohydrate antigen (galactose-α-1,3-galactose) inducing an IgE response. Later, eating meat, drinking milk, or eating butter (because the antigen is found in mammals we eat, but not humans) induces an allergic reaction causing abdominal pain and diarrhea often without other allergic manifestations [59]. However, the exact prevalence of α-gal in Rome-positive patients labeled as IBS-diarrhea remains to be determined.

## Duodenal bacteria and food

There is growing evidence of microbial disturbances in the duodenum in FD compared to healthy controls, with diet implicated in modifying small intestinal microbial composition [60–62]. Saffouri et al. found that a diet low in dietary fiber and high in refined carbohydrates reduced the diversity of the duodenal microbiota, and this was associated with increased symptom burden in individuals with DGBIs [62]. Utilizing the patented Brisbane aseptic device [63], duodenal biopsies were obtained from FD patients and controls. The dominant bacterial genus identified was *Streptococcus* by 16S, and the more bacteria present correlated with a higher symptom burden and lower quality of life [64].

Follow-up work confirmed these initial findings [61] and identified a novel *Streptococcus salivarius* in the duodenal mucosa of FD [65]. Further, those with FD and/or IBS reporting wheat induced symptoms had increased Streptococci in contrast to those without wheat sensitivity, and gut homing CD4^+^α4^+^ β7^+^CCR9^+^ T cells were also increased in those with wheat sensitivity [66]. Other evidence implicates Streptococci in digesting wheat proteins and therefore promoting presentation of antigens to the small intestinal mucosa [67, 68], leading to the hypothesis that it is the interaction of food with bacteria that may induce atypical (non-IgE) food reactions in FD and IBS.

A recent prospective study reported altered oral, duodenal, and stool microbiota in 12 individuals with FD compared to controls [69]. These alterations in duodenal microbiota were associated with habitual diet and changes to the epithelial barrier function. Participants with FD had an increased abundance of duodenal *Streptococcus* which trended positively with impaired tight junction protein expression and was significantly negatively associated with carbohydrate intake. *Butyricicoccus* was reduced in the stool of FD participants, and this was negatively associated with symptom severity and impaired tight junction expression. Furthermore, protein intake, polyunsaturated fatty acids, and valine intake were associated with improved mucosal integrity. This supports research in youth that altered lipid and amino acid metabolism is evident in individuals with FD and eosinophilia [25]. Notably, Tziatzios et al. identified differences in the duodenal microbiota between FD and controls, but not between FD and IBS, suggesting post-prandial symptoms in both conditions may be related to similar changes in the duodenal microbiota and subsequent microbial metabolite actions [60]. These data suggest diet may modulate the small intestinal microbiota which is associated with mucosal integrity, metabolite function, and symptom response.

Dietary intervention may be preferable to antibiotic treatment in conditions related to small intestinal bacterial overgrowth (SIBO). While antibiotic use has been found to be efficacious in small intestinal bacterial overgrowth, symptom relief is not sustained in a subset of patients [70]. This may be due to the broad-spectrum approach of antibiotic use and an oversight regarding the importance of bacterial synergy and community dysbiosis in determining health outcomes.

## Blocking histamine to reduce food-induced symptoms in IBS and FD

Because activated mast cells play a role in both IgE and non-IgE food-induced symptoms leading to histamine release, the concept of blocking histamine in IBS and FD is of major interest. Histamine in mouse models induces abdominal pain, and histamine release induces immune activation attracting more mast cells [71]. Duodenal mast cells are not significantly increased in FD (in contrast to IBS), but eosinophils regulate mast cell histamine release and have high histamine receptor expression [72].

Bacteria and diet may also play a role in increasing local histamine release and driving symptoms. One study identified *Klebsiella aerogenes*, present in the microbiota of up to 25% with IBS, was the main bacterial producer of histamine (via breakdown of dietary histidine) [71]. However, over 100 other bacteria are potential histamine producers via histidine decarboxylase [73] and ingestion of high histamine containing foods has been reported to induce IBS symptoms [74]. Furthermore, while the low FODMAP diet is predominantly accepted to benefit symptoms via reductions in colonic fermentation, emerging evidence suggests histamine production is reduced on a low FODMAP diet [75] and neuronal excitability is reduced in IBS pain responders either following a low FODMAP diet or taking a histamine receptor antagonist [76], potentially illuminating the histamine mediated effects of a low FODMAP diet. This is supported by observational data in 9710 participants who completed a food and symptom diary, with significant associations between symptoms of diarrhea, pain, gas and bloating and histamine, not FODMAP, containing foods [15].

In IBS, blockade of H1 receptor with ebastine in two randomized controlled trials modestly improved IBS symptoms over placebo without changing rectal visceral hypersensitivity [77, 78]. In an observational study in FD, combined H1 and H2 blockade significantly reduced dyspepsia symptoms and the reduction was greater in those with higher duodenal eosinophil counts [79]. These studies suggest a safe, cheap antihistamine may provide major symptom benefits in both IBS and FD, but this remains to be confirmed in adequately powered randomized trials. Elucidating how dietary histamine, histamine produced by gastrointestinal bacteria or released by mast cells relate to each other will likely substantially progress understanding of IBS and FD etiology and pathophysiology.

## Future research

The current evidence for the role of non-typical food allergy in DGBI is limited by difficulties in sampling techniques, variable diagnostic criteria, and reliable dietary assessment methods. In order to determine systemic effects of food intake on the immune, the nervous, and the gastrointestinal systems, study designs inclusive of all parameters of interest are necessary. This likely includes both objective and subjective outcomes including inflammatory markers and immune cell response, small and large intestinal microbiota, and associated functional data as well as patient-reported gastrointestinal outcomes.

Considerable investment of research resources into DGBI-specific altered dietary intake, specifically adaptive versus maladaptive dietary modification is warranted. This research should encompass optimal dietary and medical management of DGBI to ensure dietary therapy and management remains adaptive but appropriate. It would also incorporate screening for maladaptive food-related behaviors that are indicative of possible disordered eating or eating disorders, and pathways for diagnosis of these conditions in those with DGBI [16].

As low-grade intestinal inflammation appears to be an important area of focus in understanding how the immune system is implicated in DGBI, it is essential that the study outcomes are consistent to allow for data consolidation. For example, abnormal eosinophil cut-offs vary between studies limiting data synthesis, however recently, standards for eosinophil counts have been set and these should be adhered to in future research [28].

Both FD and IBS are positively diagnosed using ROME IV criteria. Diagnosis of FD requires upper endoscopy to rule out structural- or immune-related causes of symptoms. However, many studies in FD use inclusion criteria based on self-report without upper endoscopy. Hence, participants may not truly have FD, potentially hindering the specificity of results.

A common limitation of diet and DGBI research is subjective outcome measures. Recent advances in technologies that allow assessment of the gastrointestinal tract in real time provide exciting opportunities to better understand the effect of food in these conditions. These include CLE, high-resolution manometry, body surface gastric mapping, and magnetic resonance imaging [80]. However, careful consideration of the clinical relevance of these studies is needed. For example, for CLE studies, consideration of the state in which a dietary component is presented to the intestinal mucosa post digestion in the mouth and the small intestine should be replicated to ensure translational outcomes.

Furthermore, many of the dietary characteristics of interest here are not routinely assessed in studies collecting dietary data. National food composition databases are used to determine the macronutrient and micronutrient intake of foods reported in most of the studies included here, however these do not include potentially key food components, such as food chemicals, histamine and FODMAPS. A difficulty in the assessment of food chemicals and histamine intake is varying levels of these components in foods dependent on ripeness, food processing, and origin. Additionally, many dietary components not assessed in routine dietary assessment methods have been associated with alterations in colonic microbiota, and hence dietary assessment methods that assess these are likely relevant to studies investigating the duodenal microbiota [81].

## Implications for clinical practice

Observational studies indicate individuals with DGBI are self-restricting dietary components including wheat, histamine, food chemicals, and FODMAPS potentially without scientific merit. At present, there is limited evidence to recommend exclusion of specific dietary components to modulate the immune system in DGBI. This paper has outlined that self-reported symptom response to multiple dietary components is evident in DGBI, and that these may differ by symptom profile. These nuances may be a result of individualized immune, microbial, and clinical characteristics and as such, dietitians are well-placed to work with an individual to determine individual food triggers and dietary requirements while maintaining nutritional adequacy. Ideally, with progress in this research area, microbial and immune characteristics may be able to be used in clinical practice to predict individual dietary triggers and inform personalized dietary advice. Furthermore, this review has discussed adaptive food behaviors as a result of gastrointestinal symptoms, highlighting that clinicians must be cognizant of these risks and work within patient-centered multidisciplinary teams before implementing restrictive diets in DGBI [16]. Future research with a focus on personalized dietary therapy and appropriate differential diagnostic tools will pave the way for reduced reliance on broad untargeted elimination diets, hence reducing dietary restriction and associated risks.

## Conclusions

Current pharmaceutical treatments for IBS and FD recommended in guidelines are not optimal, with rarely more than 10% gain over placebo [1–3]. In terms of dietary therapy for DGBIs, while elimination diets (e.g., low FODMAP) are broadly effective for symptom management, mechanistic understanding of diet–gut–brain interactions is too limited to allow for personalized, treatment-focused approaches. Further, treatment failure is common, any benefit ceases on stopping therapy, and none are curative [1–3]. We would argue there is now compelling evidence to re-evaluate our current approach to management of IBS and FD in light of recent immunological and microbial discoveries.

**Fig. 1:**
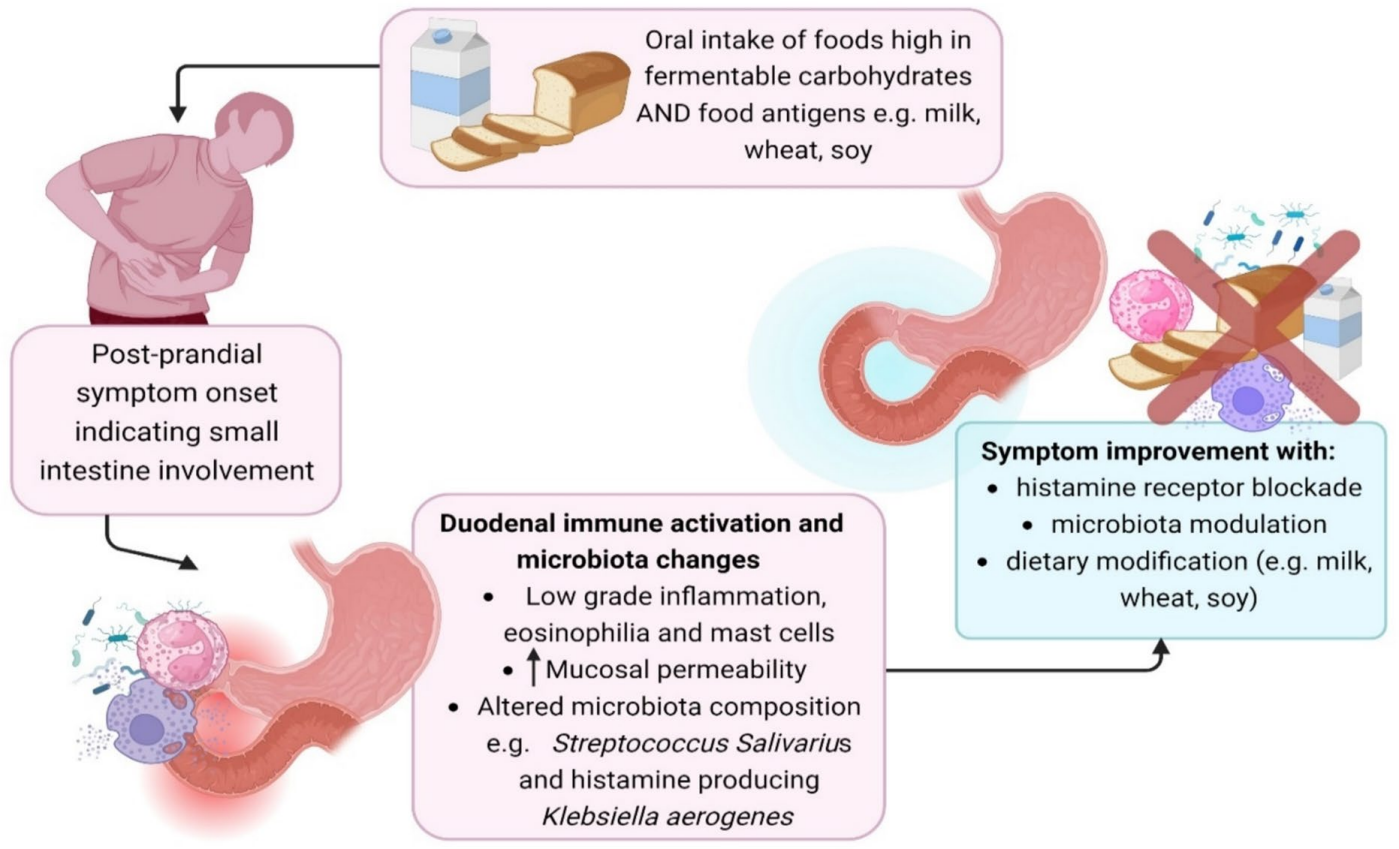
Schematic representation of possible relationship between food components, symptoms, duodenal immune activation and microbiota changes in functional dyspepsia and irritable bowel syndrome

Like in eosinophilic esophagitis, removal of food antigens may provide a symptom cure in those with FD or IBS and an atypical food allergy (Fig. 1). Indeed, in those responding to a low FODMAP diet, it may be the removal of food antigens that provide the benefit in at least some cases (and those not responding may have less common food allergic reactions not a part of the FODMAP grouping). Finally, blockade of histamine release or interruption of histamine production may also provide substantial, albeit temporary symptom relief. The fulfillment of this emerging work will likely represent a paradigm shift in explaining and managing FD and IBS.

## Abbreviations

IBS: Irritable bowel syndrome
FD: Functional dyspepsia
DGBI: Disorders of gut–brain interaction
FODMAP: Fermentable oligosaccharides, disaccharides, monosaccharides and polyols
